# High Comorbidity Burden in Patients with SLE: Data from the Community-Based Lupus Registry of Crete

**DOI:** 10.3390/jcm10050998

**Published:** 2021-03-02

**Authors:** Irini Gergianaki, Panagiotis Garantziotis, Christina Adamichou, Ioannis Saridakis, Georgios Spyrou, Prodromos Sidiropoulos, George Bertsias

**Affiliations:** 1 Department of Rheumatology and Clinical Immunology, University of Crete School of Medicine, 71500 Giofirakia, Greece; iriniger@hotmail.com (I.G.); christina.adamichou@gmail.com (C.A.); sar_giannis@outlook.com.gr (I.S.); gergianakii@gmail.com (G.S.); sidiropp@uoc.gr (P.S.); 2 Department of Rheumatology and Clinical Immunology, University Hospital of Heraklion, 71500 Heraklion, Greece; 3 Institute of Molecular Biology and Biotechnology, Foundation for Research and Technology-Hellas (FORTH), 70013 Heraklion, Greece; 4 Laboratory of Immune Regulation and Tolerance, Autoimmunity and Inflammation, Biomedical Research Foundation of the Academy of Athens, 11527 Athens, Greece; garantziotis.p@gmail.com; 5 Division of Immunology and Rheumatology, Hannover Medical University, 30625 Hannover, Germany

**Keywords:** autoimmunity, metabolic risk factors, cardiovascular, mental disorders, disease severity, social factors

## Abstract

Comorbidities and multimorbidity, often complicating the disease course of patients with chronic inflammatory rheumatic diseases, may be influenced by disease-intrinsic and extrinsic determinants including regional and social factors. We analyzed the frequency and co-segregation of self-reported comorbid diseases in a community-based Mediterranean registry of patients (*n* = 399) with systemic lupus erythematosus (SLE). Predictors for multimorbidity were identified by multivariable logistic regression, strongly-associated pairs of comorbidities by the Cramer’s V-statistic, and comorbidities clusters by hierarchical agglomerative clustering. Among the most prevalent comorbidities were thyroid (45.6%) and metabolic disorders (hypertension: 24.6%, dyslipidemia: 33.3%, obesity: 35.3%), followed by osteoporosis (22.3%), cardiovascular (20.8%), and allergic (20.6%) disorders. Mental comorbidities were also common, particularly depression (26.7%) and generalized anxiety disorder (10.7%). Notably, 51.0% of patients had ≥3 physical and 33.1% had ≥2 mental comorbidities, with a large fraction (*n* = 86) displaying multimorbidity from both domains. Sociodemographic (education level, marital status) and clinical (disease severity, neurological involvement) were independently associated with physical or mental comorbidity. Patients were grouped into five distinct clusters of variably prevalent comorbid diseases from different organs and domains, which correlated with SLE severity patterns. Conclusively, our results suggest a high multimorbidity burden in patients with SLE at the community, advocating for integrated care to optimize outcomes.

## 1. Introduction

It has long been appreciated that patients with Systemic Lupus Erythematosus (SLE) suffer from a chronic disease course burdened with comorbid conditions from multiple organs [1,2]. This was illustrated in a large case-control study utilizing data from the UK Clinical Practice Research Datalink, where SLE patients had significantly increased incidence for comorbidities with adjusted relative rates ranging from 1.31 to 7.83 [3]. These results are further supported by observational studies examining individual disorders such as infections [4,5,6], hypertension and metabolic risk factors [7,8], atherosclerotic vascular events [9,10,11,12,13], malignancies [14,15,16], and osteoporosis [17,18]. Psychiatric comorbidities, albeit less well studied, are also prevalent in SLE patients [19,20]. Notably, comorbid diseases may occur both at early and late stages of the disease, and tend to accumulate in individuals, also known as multimorbidity, which is an emerging frontier in autoimmune rheumatic diseases [21]. Compared to morbidity, multimorbidity represents a broader, patient-centered concept that extends beyond the coexistence of disorders and implies potential disease interaction and pathophysiological links [21].

Similar to other diseases, comorbidities have been shown to correlate with a number of adverse outcomes in patients with SLE including poor health-related quality of life [22,23], reduced work productivity [24], irreversible end-organ damage [25], increased hospitalizations and healthcare costs [26,27], and excess mortality [28]. Accordingly, clinical management of SLE should focus on strategies for preventing or mitigating the impact of comorbidities [1,29], also emphasized in the recommendations issued by regulatory committees such as the European League Against Rheumatism (EULAR) [30,31,32] and the American College of Rheumatology (ACR) [32].

Although incompletely understood, occurrence of comorbidity and multimorbidity might be due to the inflammatory (e.g., soluble mediators) and clinical (e.g., pain) burden of the underlying index disease (SLE), the effect of administered treatments (e.g., glucocorticoids), and shared pathogenic risk factors [33]. In this context, very few studies have examined the association between lupus activity and severity patterns and comorbidities in patients with SLE [34]. Importantly, development of comorbidities may be influenced by parameters such as race and ethnicity [35,36], access to medical care [37] and other social determinants [38]. Accordingly, examining the prevalence of comorbid diseases and their associated factors in different settings is important to obtain a comprehensive view of the clinical burden of SLE and unravel medical needs at regional level.

To this end, we recently established the Cretan Lupus Epidemiology and Surveillance Registry in order to examine SLE occurrence trends and disease characteristics in Crete, the fifth largest Mediterranean island [39,40]. This is a community-based registry of patients who reside both at rural and urban districts and receive care from the primary to tertiary level. Therefore, a wide spectrum of disease presentations is captured ranging from milder to severe forms of SLE [39,40]. Herein, we report on the frequency of comorbid diseases based on patient-reported data collected during face interviews upon enrolment to the registry, and we explore demographic and clinical variables associated with the presence of multiple comorbidities. Driven by our results showing co-segregation of comorbidities in SLE patients, we performed cluster analysis to determine phenotypes of comorbidities in our dataset. Our findings suggest a high burden of comorbid diseases and multimorbidity within SLE patients encountered at the community, related to both disease and socio-demographic characteristics.

## 2. Materials and Methods

### 2.1. Source Population and Setting

Crete is the southernmost and largest island of Greece with a relatively stable population of 623,065 inhabitants (2011 National Census). About 61% of the residents live in rural (≤10,000 dwellers) and the remaining 39% in urban (>10,000 dwellers) regions. The health system is mixed public and private, and patients can visit a specialist at the hospital or privately. The Department of Rheumatology, Clinical Immunology at the University Hospital of Heraklion (Panepistimiou, Iraklio, Greece) serves as a referral centre for patients with autoimmune rheumatic diseases, connects to private rheumatologists and general physicians working in rural health centers, and provides inpatient and outpatient services from primary to tertiary level [39,40]. Access to primary and specialized care is generally not considered to be hampered in Crete [41].

### 2.2. The Cretan Community-Based Lupus Registry

Details on the registry and its methodology are provided elsewhere [39,40]. Briefly, the main inclusion criterion was any definite or possible case of SLE aged >15 years at the time of enrolment, and the primary aim was to estimate the frequency and burden of SLE in the community. Cases were diagnosed by experienced rheumatologists and ascertained by the American College of Rheumatology (ACR) 1997 [42] and Systemic Lupus International Collaborating Clinics (SLICC) 2012 [43] classification criteria. To achieve the highest possible patient enrolment from all four prefectures of the island, from the community to the tertiary centre, and reduce the risk of selection bias for the more severe, hospital-based cases, we pursued active multisource recruitment from all hospital departments (Rheumatology, Dermatology, Nephrology; inpatient and outpatient clinics) caring for lupus patients and also, private rheumatology practices across the island. Patients were enrolled between April 2012 and December 2015 with a stable enrolment rate of 8 to 12 patients per week and an acceptance rate > 95%. Upon inclusion, patients were assessed clinically and completed a structured questionnaire regarding residential history, lifestyle factors, and disease characteristics [39,40]. A review of the medical charts, supervised by trained researchers with data cross-checking and quality control was conducted to reduce possible misclassification and information bias. Administrative data were used regarding hospitalizations. A total of 460 patients were enrolled, following informed consent, and gave face interviews except for five patients who could not stay after their visit to the outpatient clinic and were instead phone interviewed. This sample corresponds to more than half of the previously identified, community-based prevalent SLE cases in Crete (*n* = 753) [40]. In total, 61 cases were excluded as they did not fulfil the classification criteria or had incomplete information, thus resulting in a final dataset of 399 patients.

### 2.3. Variables and Comorbidities

We assessed for the presence of comorbid disease using patient-reported data obtained during the structured face interviews, as several studies have previously demonstrated high concordance between self-reported and medical record- or hospital-based comorbidities data [44,45,46,47]. Specifically, questionnaires were used to collect information on: gender, ethnicity, education level (≤ or >12 years), marital status, employment status, and descent (Cretan (for at least three past generations), other), place of current and upbringing residency and any translocations (urban-rural), smoking (current, never, ever, pack-years), body mass index (BMI; kg/m^2^), use of cosmetics and pesticides (frequent, ever, no use) [39]. The following comorbidities were also assessed by means of predefined questionnaire and were further ascertained by medical charts screening and use of relevant medications: allergies (allergic rhinitis, asthma, urticaria, drug allergies), diabetes mellitus, hypertension, dyslipidemia, thyroid disease (cancer, nodules, autoimmune thyroiditis), osteoporosis or osteoporotic fracture, heart disease, neurologic condition, cancer, kidney disease, lung disease, liver or gallbladder disease, peptic ulcer disease, blood disorders or thalassemia trait, skin diseases. Latent (tuberculosis, HIV) or recurrent urinary tract infections were assessed by self-reporting or medical charts screening and no confirmatory essay was used. The following mental conditions were assessed: depression, generalized anxiety disorder, bipolar disorder, memory and cognitive disorder, eating disorders, alcohol dependence, illicit drug dependence, suicidal attempt. The age-adjusted Charlson Comorbidity Index (CCI) [48] was calculated for each patient.

### 2.4. Clinical Data Abstracted from the Medical Records

The following data were extracted from the medical charts: clinical diagnosis and date of diagnosis, SLE classification criteria [42,43], biopsy-proven lupus nephritis, neuropsychiatric lupus (NPSLE) (defined by multidisciplinary consensus and attribution models [49]), organ damage (assessed by the SLICC/ACR damage index (SDI) [50]). For every patient, disease was categorised as mild, moderate or severe according to the British Isles Lupus Assessment Group (BILAG) classification system [51], and as previously described [39]. Briefly, the medical charts of all patients were scrutinized to detect incident activity (at any timepoint during the disease course) from individual organs and domains. Manifestations classified as “BILAG A” were assigned as severe, “BILAG B” as moderate, and the remaining ones (e.g., polyarthritis not restricting mobility and not affecting large joints; hair loss without excessive alopecia and without scalp skin inflammation; thrombocytopenia > 50,000/μL) as mild. These data were entered into a structured sheet and were collectively evaluated by two experienced Rheumatologists (G.B., C.A.) who provided their overall assessment of disease severity.

### 2.5. Statistical Analysis and Hierarchical Clustering

Data are expressed as mean (±standard deviation (SD)) or percentages as appropriate. The Student’s t-test or Mann–Whitney non-parametric test were applied for continuous, and the chi-squared or Fisher’s exact test for categorical variables. Any missing data from the questionnaires (mainly “do not know” answers) was handled by complete case analysis method. Stepwise logistic regression analysis (adjusted for possible confounding variables including age at diagnosis, disease duration, gender, smoking, residence place (urban or rural) and number of ACR-1997 criteria) was performed to examine the association between selected demographic and clinical parameters with comorbidities outcome measures (≥3 physical comorbidities, ≥2 mental comorbidities, CCI ≥1) (IBM SPSS Statistics for Mac, version 22.0. Armonk, NY: IBM Corp, Armonk, NY, USA). We created a correlation matrix for comorbidities groups (according to the affected organ or domain) and used the Cramer’s V statistic to determine the magnitude of pairwise associations; strong relationships were defined according to a threshold of V value > 0.10. Utilizing Gower’s distance and complete linkage method, hierarchical agglomerative clustering of the patients according to their comorbidity profile was performed. Clusters statistics were analyzed using the R package (version 3.5.0, R Core Team, 2018, R Foundation for Statistical Computing, Vienna, Austria). The chi-squared statistic was used to examine whether the distribution of the comorbidities differed between the identified patient clusters. The heatmap of the frequencies of comorbidities groups across the patient clusters was created using the pheatmap package (version 1.0.12, R Core Team, 2018, R Foundation for Statistical Computing, Vienna, Austria).

## 3. Results

### 3.1. High Prevalence of Comorbid Diseases in SLE Patients at the Community

We studied 399 SLE patients (91.2% females) with an average disease duration of 10 years. At the time of assessment, approximately 11% had history of biopsy-proven nephritis and a similar proportion had neuropsychiatric disease attributed to SLE (Table 1).

Among the most prevalent physical comorbidities were thyroid (45.6%) and metabolic disorders (hypertension: 24.6%, dyslipidemia: 33.3%, obesity: 35.3%), followed by osteoporosis (22.3%) and cardiovascular diseases (20.8%) and allergic disorders (20.6%) (Table 2). Mental disorders were also common (45.1%) particularly depression (26.7%) and generalized anxiety disorder (10.7%). Female SLE patients had significantly increased frequency of thyroid diseases (51 vs. 16%, *p* < 0.001), allergic diseases (21 vs. 3%, *p* = 0.006), and osteoporosis (19 vs. 6%, *p* = 0.05) compared to male patients, whereas respiratory comorbidities (21 vs. 9%, *p* < 0.001) and alcohol abuse (3 vs. 0%, *p* < 0.01) were more prevalent among male patients.

Overall, SLE patients had an average (±SD) 2.8 (±2.0) physical and 0.9 (±1.3) mental comorbidities with a mean age-adjusted CCI of 0.91 ± 1.16 (Table 3). Notably, 51.0% of patients had three or more physical comorbidities and 33.1% had two or more mental comorbidities, suggesting a high comorbidity burden in patients with SLE at the community.

### 3.2. Co-Segregation of Physical and Mental Comorbidities in Patients with SLE

**Figure 1:**
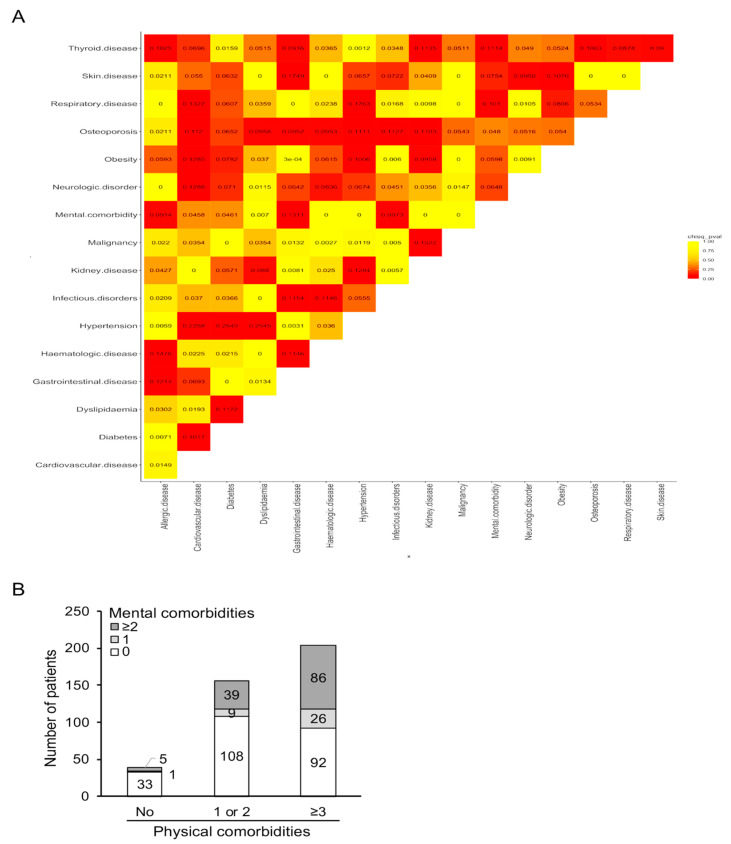
Co-segregation of comorbidities in patients with SLE at the community-based registry of Crete. (**A**) Correlation matrix of various comorbidities from diverse organs and domains. Numbers inside each box represents the Cramer’s V statistic estimated for each pair of comorbidities with values > 0.10 signifying robust correlation. Color intensity corresponds to the chi-squared *p*-value for each pairwise association. (**B**) SLE patients were categorized according to the number of physical (none, 1 or 2, ≥3) and mental (none, 1, ≥2) comorbidities as described in the main text. Y axis shows the number of patients with various combinations of physical and mental comorbid disorders.

Driven by our findings, we carried out a correlation analysis to detect concurrent comorbidities. We noted several pairs of diseases with high prevalence in our sample, for instance, thyroid disease and dyslipidemia (*n* = 71, 18%), dyslipidemia and hypertension (*n* = 59, 15%), allergic disorders and thyroid disease (*n* = 49, 12%), gastrointestinal disorders, and thyroid disease (*n* = 45, 11%). Mental disorders (merged together into a single group) often coexisted with thyroid disease (*n* = 94, 24%), dyslipidemia (*n* = 62, 16%), and gastrointestinal disorders (*n* = 45, 11%). Next, the Cramer’s V statistic was implemented to obtain a statistically robust measure of the relative strength of association between different pairs of comorbidities. We observed a substantive relationship between allergic and hematological diseases, hypertension and diabetes, dyslipidemia, and cardiovascular diseases, the latter concurring also with other comorbidities such as obesity, osteoporosis, neurologic, and respiratory disorders (Figure 1A). Other associations included thyroid disease with kidney and mental disorders, skin with gastrointestinal and neurological diseases, kidney disease with hypertension. By further examining the co-segregation of comorbidities, we found a positive correlation between an increasing number of physical and mental disorders. Specifically, within SLE patients with ≥3 physical comorbidities, a large fraction (*n* = 86) also reported multiple (≥2) mental disorders (Figure 1B). Conversely, within 39 patients with no physical comorbidity, only 5 had two or more mental disorders. Together, these results suggest a substantial multimorbidity burden in SLE patients including the co-existence of physical and mental disorders.

### 3.3. Predictors of Morbidity and Multimorbidity in Patients with SLE

We evaluated for factors associated with comorbidities in our SLE dataset by analyzing for the presence of multiple physical comorbidities (≥3), mental comorbidities (≥2), or CCI ≥ 1. Demographic, social and clinical features were treated as independent variables initially at univariate level, followed by multivariate logistic regression. We found that moderate vs. mild disease was associated with increased risk (adjusted odds ratio (OR) 2.30) and that higher vs. lower (≥12 years vs. <12 years) education level was associated with decreased risk (OR 0.46) for physical multimorbidity (Table 4). Disease duration correlated with the total number of physical comorbidities (Spearman’s rho 0.152, *p* = 0.019) but only at univariate level. In the case of mental multimorbidity, independent predictors were the marital status (divorced or widowed patients having OR 2.76) and the ACR 1997-defined neurologic disease item (OR 6.02). Finally, morbidity defined according to CCI ≥1 was associated with the education level, marital status, and number of ACR 1997 classification criteria (OR 1.30 per 1-item). In a separate analysis, we also correlated the total number of used (ever) immunosuppressive or biological agents (azathioprine, mycophenolate, belimumab, cyclophosphamide, rituximab) with the sum of physical -but not mental- comorbidities (Spearman’s rho 0.136, *p* = 0.036) and adjusted Charlson Comorbidity Index (Spearman’s rho 0.183, *p* = 0.001). Altogether, both sociodemographic and clinical factors are linked to the comorbidities risk in patients with SLE at the community.

### 3.4. Distinct Comorbidities Phenotypes in Patients with SLE Revealed by Cluster Analysis

**Figure 2:**
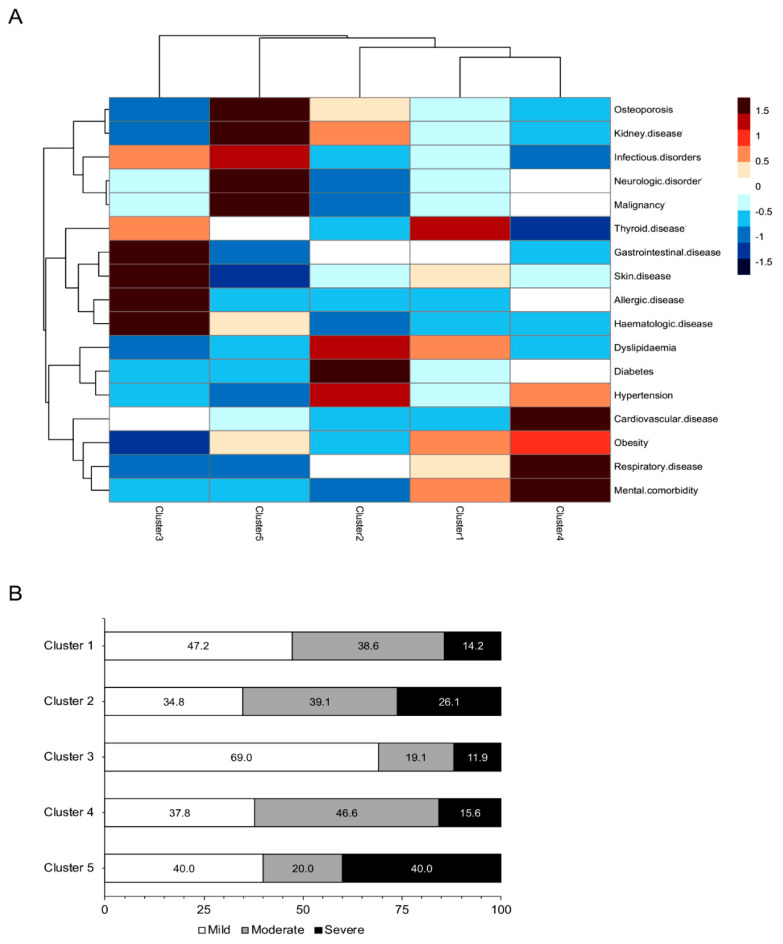
Distinct comorbidities phenotypes in patients with SLE revealed by cluster analysis. (**A**) Utilizing Gower’s distance and complete linkage method, hierarchical agglomerative clustering of the patients according to their comorbidity profile was performed. The chi-squared statistic was used to examine whether the distribution of the comorbidities differed between the identified patient clusters. The heatmap of the frequencies of comorbidities groups across the patient clusters is shown. Legend depicts the relative frequency (ranging from −1.5 to +1.5) of each comorbidity within each cluster. (**B**) Prevalence of SLE severity patterns (mild, moderate, severe) based on the BILAG system across the identified comorbidities clusters (cluster 1 to 5). Numbers are proportions (%).

We next examined whether SLE patients can be classified into distinct phenotypic groups as this may further enhance our understanding of the complexity of comorbid diseases beyond a single-disease perspective, and how these might differ according to demographic, clinical, or other determinants. Hierarchical agglomerative clustering revealed five patient clusters, each with variable prevalence of comorbid diseases from various organs and domains (Figure 2A). Cluster 1 included the majority of patients (*n* = 227) and was characterized by increased prevalence of thyroid disease and to lesser extent, obesity, dyslipidemia and mental comorbidities. Cluster 2 (*n* = 46) had high frequency of metabolic risk factors, cluster 3 (*n* = 43) of gastrointestinal, skin, allergic, and hematologic diseases, and cluster 4 (*n* = 45) of metabolic risk factors, cardiovascular, respiratory, and mental disorders. Cluster 5 included a minority (*n* = 6) of SLE patients with relatively increased prevalence of osteoporosis, malignant, neurologic, infectious, and kidney disorders. Identified clusters did not differ in terms of gender, residence place, education level, smoking status, age of SLE diagnosis, and disease duration. Notably, clusters 2 and 5 included patients with high frequency of biopsy-proven nephritis (28.3 and 33.3%, respectively) as compared to other clusters (*p* < 0.001). NPSLE had highest prevalence in clusters 4 and 5 (20.0 and 66.7%) than in clusters 1–3 (*p* < 0.001). Prevalence of combined lupus skin lesions (both malar and discoid rash) was higher in cluster 1 (7.6%) as compared to clusters 2 (4.5%), 3 (2.3%) and 4–5 (0.0%) (*p* = 0.019). No associations were detected with regards to other clinical or immunological disease features. Patterns of disease severity differed across the aforementioned groups with clusters 1 and 3 including increased fractions of patients with mild disease (47.2 and 69.0%, respectively) as compared to clusters 2 and 5 which comprised severe SLE patients (26.1 and 40.0%, respectively) (*p* = 0.024) (Figure 2B).

### 3.5. Association between Morbidities and Clinical Outcomes in Patients with SLE

Previous work has demonstrated that comorbid diseases may have an adverse impact on miscellaneous patient and disease outcomes in SLE. Analysis of our data did not reveal a statistically significant association between physical or mental multimorbidity and rates of irreversible organ damage (SDI ≥ 1), although the total number of physical comorbidities showed a trend for correlation with the SDI (Spearman’s rho = 0.126, *p* = 0.050). Notably, patients with ≥3 physical comorbid diseases had experienced an increased number of hospitalizations due to active SLE (1.96 ± 0.40 vs. 0.91 ± 0.17 in counterparts with 0–2 morbidities, *p* = 0.018).

## 4. Discussion

Herein, we evaluated the presence of comorbid diseases and their determinants in SLE patients from a homogenous south European population. Background, race and ethnicity, and geographical characteristics are important determinants of both lupus ominosity and associated comorbid disorders [7,35,36,52,53]. Our findings, derived from a community-based, Caucasian registry that captures both severe and milder disease forms, highlights a high burden of physical and mental comorbidities among SLE patients, which tend to co-segregate and cluster into distinct phenotypic groups. Moreover, we demonstrate that comorbidities may be associated with clinical disease severity and certain sociodemographic factors, which further supports the complex nature of comorbidity in SLE and the need for holistic approach.

Our results reiterate the previously reported prevalence of numerous comorbid medical disorders in patients with SLE. Although direct comparisons are hampered due to differences in the study design, assessment and documentation methods, and the definitions used, our findings concord with published data underpinning increased occurrence of metabolic and atherosclerotic factors such as hypertension, dyslipidemia, obesity, diabetes, cardiovascular, and cerebrovascular disease as compared to the general population [7,8,9,10,11,12,13,54,55,56,57,58]. Cardiovascular burden is also increased in SLE, attributable to the interplay between demographic (e.g., gender, ethnicity), disease duration, traditional risk factors (including smoking), lupus autoimmunity such as type I interferon signaling, and the known deleterious effects of chronic glucocorticoids use [7,56,58,59,60,61,62,63]. In line with this, circumstantial non-randomized evidence suggests that attainment of low disease activity state on a minimal background dose of glucocorticoids is associated with reduced risk for cardiovascular events in SLE [64].

Disorders of the thyroid gland comprised a prevalent condition in our cohort, consistent with case-control studies indicating a statistically significant association between SLE and autoimmune and non-autoimmune thyroidopathies [65,66,67]. Moreover, osteoporosis, a well-established comorbidity in SLE [17,18,68] attributed to multiple factors [69], was reported by a high proportion (22.3%) of our patients. Another notable finding was the high frequency of allergic disorders (20.6%), which aligns with epidemiological evidence demonstrating that SLE patients have an estimated 1.4 to 2.3-fold increased risk for atopic dermatitis, allergic rhinitis, allergic conjunctivitis, or asthma [70,71]. Intriguingly, lupus and allergy share—to some extent—common genetic predisposition, environmental factors, and immune pathways such as increased serum immunoglobulin E levels and mast cell activation [72]. Malignant disorders are also more prevalent in SLE patients than the general population [3].

Besides physical comorbidities, a considerable number of patients in our study reported at least one mental comorbidity, in particular depression and anxiety. This concords with observational studies underlining increased prevalence of the aforementioned conditions and also, cognitive complaints, in SLE patients as compared to the general population [20,73,74,75,76]. Other psychiatric disorders including bipolar [77], suicidal ideation [78], schizophrenia [79], and sleep disturbances [80], are also encountered more frequently in SLE. To this end, fibromyalgia, which was not evaluated in our cohort, represents a common SLE comorbidity often correlating with or considered to be part of the spectrum of mental conditions [19]. Although these comorbidities are typically not attributed to direct immune insult against the central nervous system as in the case of primary neuropsychiatric SLE [49], nonetheless circumstantial evidence implicates soluble inflammatory mediators in their pathogenesis [81,82].

The multimorbidity state has been well documented in inflammatory arthritides, such as rheumatoid arthritis [21,83,84,85], whereas fewer reports exist in SLE [86,87]. A remarkable finding in our study was the high prevalence of multimorbidity with more than half of SLE patients reporting ≥3 physical and about one-third of patients reporting ≥2 mental comorbid diseases. In addition, we noted a positive correlation between physical and mental multimorbidity, with their synchronous presence in 21.6% of cases. Besides the apparent impact on patient well-being, the possible implication of multimorbidity on the management and outcomes of SLE remain to be determined. To this end, our analysis showed increased rates of hospitalizations due to SLE among patients with multiple physical comorbid disease, a finding that warrants further investigation in prospective studies.

Identification of morbidity predictors is valuable for recognizing high-risk patient groups and thus, adjusting medical care accordingly. Rather than focusing on isolated diseases, we analyzed for factors associated with physical and mental multimorbidity as well as CCI-defined morbid status. We found that increasing SLE severity, reflected in a BILAG-based classification of manifestations or the number of ACR 1997 classification criteria, correlated with increased odds for physical morbidity, thus corroborating evidence on the effects of high disease activity and medications (e.g., glucocorticoids) on the development of comorbid disorders [7,34,60,61,88]. This finding might be mirrored by the positive correlation between exposure to immunosuppressive and biological agents and physical comorbidities, although drug-intrinsic effects cannot be ruled out. Notably, and in agreement with studies in other patient populations [38,89,90], higher education level was linked to reduced comorbidity risk, presumably due to increased awareness, treatment adherence and better overall management of the index disease (SLE) and its complications. In the case of mental multimorbidity, neurological disorder (ACR 1997-defined) and the marital status were independent predictors. Whether the former association corresponds to increased neuropsychiatric or general inflammatory burden of SLE or is confounded by other factors is unknown. Likewise, the increased risk in widowed and separated patients could possibly be related to stressful life events or other socio-economic parameters not evaluated in the present analysis.

In view of our results demonstrating aggregation of multiple disorders from diverse organs and domains, we performed clustering analysis searching for phenotypic subgroups of clinical relevance [85,91]. Using this approach, our SLE sample was categorized into five clusters of highly coinciding comorbidities. The clusters did not differ with regards to the distribution of age, gender, other demographic and clinical factors, implying that they might be driven by other factors. Nonetheless, cluster 1, encompassing common disorders (thyroid, obesity, dyslipidemia, mental) was most prevalent in SLE patients especially those with milder disease forms. Pending confirmation in additional studies with larger patient cohorts, these findings could be helpful in the context of personalized medical care and to unravel shared underlying genetic or pathogenic links.

A number of limitations need to be acknowledged such as the fact that our study was not designed to address in detail the frequency of comorbidities and that it did not include a control group. Nonetheless, information was collected using pre-specified forms during face interviews of patients enrolled in the registry. Although self-reported and medical record- or hospital-based comorbidities data seems to correlate well [44,45,46,47], some information or misclassification errors may have occurred. As we did not monitor patient exposure to glucocorticoids, we were not able to differentiate steroid-related vs. unrelated mental disorders. Additionally, despite the fact that our sample size (*n* = 399) is generally considered sufficient, it may be underpowered to detect or evaluate infrequent disorders, differences according to gender or the autoantibodies status. To this end, our goal was not to provide an exhaustive description of isolated diseases but rather, to gain an overview of the burden of comorbidity and multimorbidity in our setting.

In conclusion, our results from a community-based, Caucasian registry highlight a considerable burden of physical and mental comorbidities in patients with SLE. Multimorbidity is a pervasive characteristic correlating both with the disease severity and sociodemographic factors such as the education and marital status, thus underscoring the need to address these factors in patient risk stratification and management. Cluster analysis of comorbidities enables the identification of distinct clinical phenotypes, which might reflect different pathophysiological processes linked to the disease. Altogether, these findings have potential implications for rheumatologists and other disciplines involved in the care of SLE patients, and emphasize the need for integrated action plans to optimize disease outcomes.

## Figures and Tables

**Table 1:** Demographic and clinical characteristics of Systemic Lupus Erythematous (SLE) patients (*n* = 399) at the time of enrolment.

Parameter	No. (%) or Mean ± Standard Deviation
Gender (female)	364 (91.2%)
Age at diagnosis (years)	42.8 ± 14.6
Disease duration (years)	9.9 ± 6.6
No. ACR criteria	4.7 ± 1.2
Lupus nephritis (biopsy-proven)	45 (11.3%)
Neuropsychiatric lupus	43 (10.8%)
Disease severity ^1^	
Mild	190 (47.7%)
Moderate	144 (36.1%)
Severe	65 (16.2%)
Organ damage	144 (36.2%)
Residence	
Rural	172 (43.1%)
Urban and semi-urban	227 (56.9%)
Education level	
<12 years	284 (71.2%)
≥12 years	115 (28.8%)
Marital status	
Single	55 (13.7%)
Married	299 (74.9%)
Divorced or separated	21 (5.3%)
Widowed	24 (6.1%)
Tobacco use	
Never	208 (55.3%) ^2^
Past	55 (14.6%)
Active	113 (30.1%)

^1^ See Materials and Methods for details on the definitions. ^2^ Data available in *n* = 376 patients.

**Table 2:** Prevalence of comorbid diseases in SLE patients at the community-based registry in Crete (*n* = 399).

Comorbidiy	Prevalence
Thyroid disease ^1^	45.6%
Mental disorder ^2^	42.1%
Depression	26.7%
Anxiety disorder	10.7%
Obesity ^3^	35.3%
Dyslipidemia	33.3%
Hypertension	24.6%
Osteoporosis and osteoporotic fracture	22.3%
Cardiovascular disease ^4^	20.8%
Allergic disorders ^5^	20.6%
Gastrointestinal disease ^6^	19.0%
Infectious disease ^7^	12.8%
Neurologic disease ^8^	10.3%
Cerebrovascular disease	2.5%
Kidney disease	9.5%
Respiratory disease	9.3%
Diabetes mellitus	8.8%
Malignant disease	4.8%
Skin disease	3.3%
Hematologic disease	2.3%

^1^ Including autoimmune thyroiditis, hypo- or hyperthyroidism, thyroid nodules; ^2^ including bipolar disease, cognitive impairment, generalized anxiety disorder, major depression, alcohol dependence, eating disorder, suicidal attempt; ^3^ defined as body mass index ≥ 30 kg/m^2^; ^4^ including coronary heart disease (angina, myocardial infarction, or coronary revascularization procedure), valvular disease, peripheral vascular disease; ^5^ including allergic rhinitis, asthma, urticaria; ^6^ including liver, gallbladder or biliary tract disease, gastroesophageal reflux disease, peptic ulcer disease, chronic diarrhea; ^7^ including latent hepatitis or HIV infection, recurrent urinary tract infections, chronic osteomyelitis; ^8^ including epilepsy, stroke, cerebrovascular disease, or other neurological diseases.

**Table 3:** Burden of comorbidities in SLE patients at the community-based registry in Crete.

Comorbidities	Mean ± Standard Deviation or Prevalence (%)
Physical comorbidities	2.8 ± 2.0
None	9.8%
1 or 2	39.2%
≥3	51.0%
Mental comorbidities	0.94 ± 1.25
None	57.9%
1	9.0%
≥2	33.1%
Charlson Comorbidity Index (CCI)	0.91 ± 1.16
>0	50.3%

**Table 4:** Predictors for the presence of comorbidities in patients with SLE.

Dependent Variable ^1^	Predictor (s)	OR (95% CI) ^2^	*p* Value
≥3 physical comorbidities	Education level		
	≥12 years vs. <12 years	0.46 (0.28–0.75)	0.002
	SLE severity		
	Moderate vs. mild	2.30 (1.43–3.71)	0.001
	Severe vs. mild	1.30 (0.71–2.41)	0.398
≥2 mental comorbidities	Marital status		
	Divorced or widowed vs. single or married	2.76 (1.43–5.35)	0.003
	ACR-1997 neurologic item		
	Present vs. absent	6.02 (1.86–19.53)	0.003
Charlson comorbidity index ≥ 1	Education level		
	≥12 years vs. <12 years	0.52 (0.31–0.86)	0.011
	No. ACR-1997 criteria (per 1-item)	1.30 (1.06–1.59)	0.013
	Marital status		
	Divorced or widowed vs. single or married	2.18 (1.02–4.68)	0.045

^1^ Backwards elimination model. Possible predictors included: age at diagnosis, disease duration, gender, smoking, residence place, number of ACR-1997 criteria; ^2^ Odds ratio (95% confidence interval).

## Data Availability

Data are available upon reasonable request.
